# THE TOLL OF BINGE EATING ON AGING: THE PHYSICAL FUNCTIONING OF OLDER WOMEN WITH WEEKLY BINGE EATING

**DOI:** 10.1093/geroni/igac059.1745

**Published:** 2022-12-20

**Authors:** Savannah Hooper, Victoria Marshall, Preston Bridges, Sara Espinoza, Pamela Keel, Andrea LaCroix, Nicolas Musi, Lisa Kilpela

**Affiliations:** UT Health Science Center San Antonio, San Antonio, Texas, United States; UT Health Science Center San Antonio, San Antonio, Texas, United States; The University of Texas Health Science Center San Antonio, San Antonio, Texas, United States; Sam and Ann Barshop Institute, UT Health San Antonio, San Antonio, Texas, United States; Florida State University, Tallahassee, Florida, United States; University of California, San Diego, La Jolla, California, United States; UT Health Science Center San Antonio, San Antonio, Texas, United States; UT Health Science Center San Antonio, San Antonio, Texas, United States

## Abstract

Binge eating (BE) is defined as eating an unusually large amount of food while feeling a loss of control; BE prevalence rates range from 12-26% in older adult women. BE is closely linked with obesity, depression, and poor micronutrient intake, as well as with cardiovascular issues and pain in younger populations. Among older adults, such morbidities can negatively affect quality of life and mobility. Yet, little is known about the toll of BE on the aging body in terms of physical function. Thus, the current investigation assessed the physical functioning of older women (60+ years) with clinical levels of BE (i.e., ≥weekly BE). Participants (N = 21) underwent several physical function tests including lower extremity functioning (Short Physical Performance Battery; SPPB), endurance (Six-Minute Walk Test), and grip strength. The majority of participants (76%) had a BE age of onset in midlife or later (age 42+). The average BMI was 35.08±8.64, and 90.48% met criteria for overweight or obesity. On the SPPB, 47.62% scored < 10, suggesting mobility limitations and predicting all-cause mortality. Most women (90.48%) scored >1SD below the age/gender matched norm on the Six-Minute Walk Test (*M*=355.96±67.04) while 61.9% scored >1SD below the norm for grip strength (*M*=24.91±6.40). Results indicate that older women struggling with BE also demonstrate diminished physical functioning, which confers risk for future health problems and poorer quality of life. Our findings suggest that intervention development for older populations struggling with BE may need to incorporate physical rehabilitation to promote healthy aging in this population.

